# Microbiological Quality of Salads Served along with Street Foods of Hyderabad, India

**DOI:** 10.1155/2014/932191

**Published:** 2014-05-14

**Authors:** Alekhya Sabbithi, R. Naveen Kumar, L. Kashinath, V. Bhaskar, V. Sudershan Rao

**Affiliations:** ^1^Food and Drug Toxicology Research Center, National Institute of Nutrition, Indian Council of Medical Research (ICMR), Jamai Osmania, Tarnaka, Hyderabad 500007, India; ^2^Statistical Division, National Institute of Nutrition, Indian Council of Medical Research (ICMR), Hyderabad, India

## Abstract

A study has been done to analyse the microbiological quality of salads served along with street foods of Hyderabad. A total of 163 salad samples, 53 of carrot and 110 of onion samples, were collected from four different zones of Hyderabad. About 74% and 56% had *Staphylococcus aureus* in carrots and onions, respectively. Fifty-eight percent of carrots and forty-five percent of onions samples contained *Salmonella*, 68% of carrots and 24% of onions had *Yersinia*. HACCP study was carried out with 6 street food vendors to identify the source of *Salmonella* contamination in salads. Food handlers were found to be responsible for *Salmonella* contamination in salads. The present study revealed the potential hazards of street vended salad vegetables, considering the handling practice usually carried out by vendors. Ninety-eight percent of the vendors did not wash the vegetables before processing and serving while about 56.6% of the vendors did not peel the vegetables. Majority of street vendors' nails were uncut. A significant difference (*P* < 0.01) was observed in *Yersinia* spp. and *Salmonella* spp. in wet-dirty chopping board when compared to clean-dry chopping board. A significant difference (*P* < 0.05) of *Staphylococcus* spp. was observed when the status of cleaning cloth was neat/untidy.

## 1. Introduction

Street vended food (SVF) includes food and beverages that are prepared and sold outdoors or in public spaces by street merchants for consumption at the location or later without further preparation [1]. Quality and safety are two common concerns with regard to street foods. Poor hygiene and sanitation practices are one of the major bottlenecks in street food vending [2].

Salad can be defined as a food made primarily of mixture of raw vegetables and/or fruits [3]. The possibility of foodborne diseases is more when salad vegetables are consumed without any heat treatment, sometimes without washing and peeling [4]. Water used for rinsing the vegetables and sprinkling to keep them fresh is also a source of contamination [5]. Fresh vegetables and fruits become contaminated with microorganisms during production, harvest, packing, and distribution [6].

Ready-to-eat fruit and vegetables requiring minimal or no further processing prior to consumption have been implicated as vehicles for transmission of infectious microorganisms of foodborne outbreaks related to gastrointestinal illness associated with fruit and vegetables [7]. In this context the present study was undertaken to analyse the microbiological quality of salads served along with street foods of Hyderabad city.

## 2. Materials and Methods

### 2.1. Study Area

The Study was carried out in Hyderabad which is the capital of Andhra Pradesh, India. The twin cities of Hyderabad and Secunderabad come under ambit of a single municipal unit, the Greater Hyderabad Municipal Corporation. For administrative purpose Greater Hyderabad Municipal Corporation was divided into many zones. Random sampling procedure was adopted to select four zones (Alwal: east zone, Yousufguda: west zone, Old City: south zone, and Secunderabad: north zone). The sample required for the study was obtained using proportionate representation according to size.

### 2.2. Questionnaire

Personal interview of the street vendor was taken and flow chart was prepared from which critical control points of contamination were examined.

Data on salad preparation, handling, and storage practices was collected using structured questionnaire that had both observational and responsive questions. Equipment used for the preparation of salad, source of water for both utensil cleaning and vegetables, utensil cleaning methods, and hand washing were considered. Salad preparation, handling practices, storage of leftover salad, storage length, and salad storage practices were observed and noted. Information on status of the premises, storage conditions for salad before cooking, cutting and chopping place, status of the serving plate, peeling and cleaning of vegetables, cleanliness of the cloth and clothing used, provision for waste disposal, presence of rodent droppings in the outlet, and exposure to insects was gathered.

### 2.3. Hazard Analysis Critical Control Point (HACCP)

HACCP was carried outin order to understand the critical points for the contamination; information on source of purchasing of salad vegetables, their transportation, storage, and further processing before consumption was obtained through personal interviews of the street food vendors (*n* = 53). Based on this information the process flow diagram was constructed. HACCP is a system which identifies, evaluates, and controls hazards which are significant for food safety. The hazard analysis includes observing salad preparation and practices to identify the sources and modes of contamination. Preparation of process flow diagram is a prerequisite for carrying out HACCP.

Samples of hand washings, knife swab, and chopping board swab were collected from six street food vendors (*n* = 6) and examined for presence of food pathogens.

### 2.4. Sample Collection and Processing

A total of 163 samples were collected from four different zones of Hyderabad. Samples were transported to the laboratory in aseptic condition. The polythene zip lock bags with salad samples were kept in an ice box maintained at 6–10 degree centigrade and processed within 2–4 hrs. Twenty-five grams of each salad sample was weighed and transferred to 225 mL of sterile buffered peptone water. The diluents of buffered peptone water were then inoculated onto the respective media.

### 2.5. Identification and Enumeration

Identification and enumeration of foodborne pathogens (*S. aureus, Salmonella *spp., and* Yersinia enterocolitica*) were performed as described by standard methods of bacteriological analytical manual. After thoroughly mixing the food sample (25 g) in buffered peptone water (225 mL) by 1 : 10 dilution, the diluents were inoculated onto their respective selective media such as MSA (mannitol salt agar) for* Staphylococcus aureus *with small round pale colored colonies, XLD (xylose lysine deoxycholate agar) for* Salmonella *spp. with red colored colonies with black centre, and YSA (*Yersinia* selective agar) for* Yersinia *spp.with transparent colonies with pink centre. After inoculation the plates were incubated for 24 hrs at 37°C in incubator. Morphological tests such as grams staining and motility tests (coagulase test for* S. aureus*) were conducted followed by biochemical tests using Hi assorted biochemical test kit (A combination of 12 tests for identification of gram-negative rods. Kits contain sterile media for citrate utilization, lysine utilization and ornithine utilization, urease detection phenylalanine deamination (TDA), nitrate reduction, H_2_S production test, and 5 different carbohydrates for utilization test: glucose, adonitol, lactose, arabinose, and sorbitol) followed by latex agglutination test kit supplied by HiMedia.

### 2.6. Statistical Analysis

The analysis was carried out by descriptive analysis (mean, minimum, and maximum) for each category of the group. Difference between the groups was tested by nonparametric Kruskal-Wallis ANOVA considering the heterogeneity of variance. Individual pair difference was tested by Mann-Whitney *U* test (SPSS-14.8 windows version was used).

## 3. Results

Microbiological analysis of salads indicated that both onion and carrot were contaminated with three pathogens.* Salmonella *was present in 58% in carrots and 45% of onion samples. Compared to onions, carrots harboured higher percentage of contamination (Table 1). Among the carrot samples procured from four zones of Hyderabad, Old City, that is, south zone, has shown high levels of contamination compared to other zones. The mean concentration ranges of* Staphylococcus*,* Salmonella*, and* Yersinia *in carrot and onion are shown (Table 2).

Seventy-six percent of onion samples from east zone had* Staphylococcus* which is high when compared to other zones.* Salmonella *was not detected in onion samples of east zone. In Old City, that is, south zone, 76% of onion samples had* Salmonella *which is high when compared to other zones (Figure 2). Samples of carrots have high levels of contamination when compared to onions.

**Figure 1:**
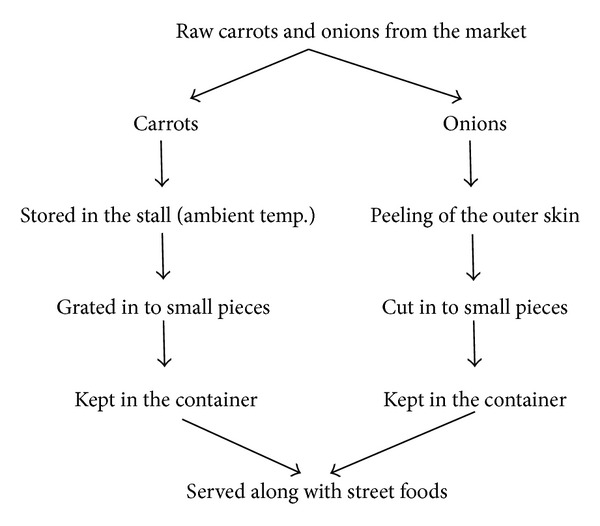
Process flow diagram for onions.

**Figure 2:**
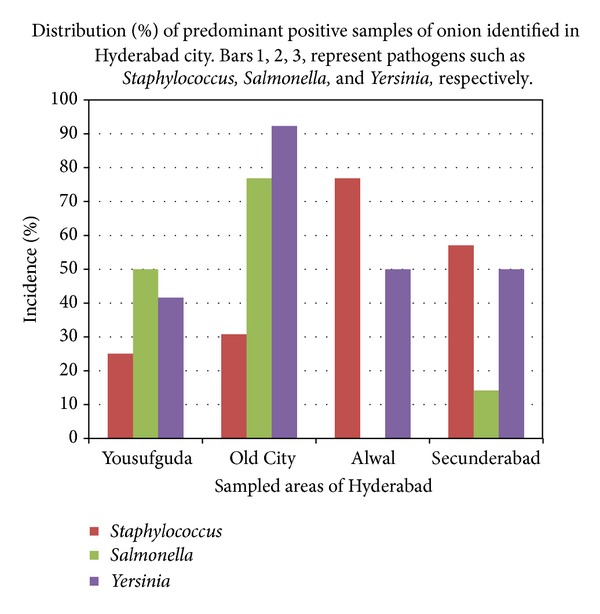
Prevalence of foodborne pathogens in onion samples of Hyderabad city.

Process flow diagram of onion and carrot indicated that, after peeling the outer skin of the onions, they were directly cut into small pieces and served without washing them. It was observed that the peeling which is very important step in processing of carrots was not done; they were directly grated and served (Figure 1). HACCP study on contamination of* Salmonella* in salads indicated that food handlers were responsible for* Salmonella* contamination (Table 4).

Most of the vendors got vegetables from whole sale market and 56% of the vendors stored them in their house and 44% of them stored it in the stall itself. About 98% of the vendors did not wash the vegetables before processing and 56% of the vendors did not peel carrots before cutting. Ninety-two percent of the vendors used knife to cut the vegetables out of which 77% of the knives were wet and dirty and 88% of the vendors did not wash knife frequently. About 54% of the vendors kept salads in open air without covering them properly. Ninety percent of them used wooden board for cutting and chopping vegetables. About 90% of the vendors' cleaning cloths were untidy and none of them wore gloves before serving salads. There were about 60% of the vendors whose nails were uncut. About 64% of the stalls are adjacent to road (Table 3).

## 4. Discussion

The study demonstrated that salads served along with popularly sold street foods are contaminated with one or many pathogens. A recent foodborne disease outbreak during 2011 in Europe and Germany was mainly due to contamination of salads such as cucumber, tomatoes, and lettuces by harmful strain of* E. coli O104:H4* [8].* Salmonella *and* Staphylococcus aureus *were the other major pathogens found in salads. A study conducted in Dehradun, India, has shown that different types of salads such as carrot, radish, tomato, coriander leaves, turnip, onion, cucumber, and beetroot were contaminated with* Salmonella* (32.28%),* E. coli* (33.14%),* S. aureus* (50.57%), and* P. aeruginosa* (60.85%) [9].

In this present study carrot and onion were chosen because they are the most commonly served salads in street foods of Hyderabad. About 74% and 56% tested positive for* Staphylococcus *in carrots and onions, respectively. About 58% of carrots and 45% of onions contained* Salmonella* and 68% of carrots and 24% of onions had* Yersinia*. Type of pathogens reported in salads, specifically with carrot, was* E. coli *O157:H7,* Salmonella *spp., and* L. monocytogenes *and for onions was* E. coli *and* Salmonella typhimurium *[10]. The presence of microbes in salads can be linked to a number of factors such as improper handling and processing, use of contaminated water during washing and dilution, cross-contamination from rotten vegetables, or the use of dirty processing utensils like knife and trays [11, 12]. The present study indicated that the bacterial load of carrots was high when compared to onions because carrots were not peeled. Peeling as expected reduces the bacterial load; a study done by Ankitha et al. indicated that a reduction of about 40% of bacterial load was observed in peeled carrot [3].

The contamination by* Staphylococcus aureus *may be due to its carriage in nasal passages of food handlers or infected workers [13]. The contamination of* Salmonella *spp. in vegetables was due to washing of vegetables with contaminated water and handling of vegetables by infected workers, vendors, and consumers in the market place which helps spread pathogenic microorganisms. HACCP study revealed that raw vegetables (carrot and onion) themselves carried pathogens and since they were not washed they continued to be present at the time of consumption. Hand washings also contained* Salmonella* which indicate that even food handlers contaminate the salads.

Previous studies have demonstrated that* Salmonella *cross-contamination occurs frequently through the use of contaminated vegetables that are improperly cleaned and undisinfected [14–16]. The serving stage is a critical point in the street food vending. Poor personal hygiene often facilitates transmission of 60 pathogens via food to humans [17]. Most of the vendors were not using hand gloves while preparing and serving street food. Vendors were carriers of a variety of bacterial enteropathogens, including* S. typhimurium *[18]. Similarly* Escherichia coli *was detected in hand washings of high income and low income mothers in India at levels of 7.0 ± 4.2 log 10 cfu/mL and 9.0 ± 5.7 log 10 cfu/mL, respectively [18]. A significant difference (*P* < 0.01) was observed in load of* Salmonella *spp. in wet, dirty chopping board when compared to the load of* Salmonella *in clean and dry chopping board. Similarly the load of* Yersinia* spp. was high in wet, dirty chopping board when compared to the load of* Yersinia *spp. in clean and dry chopping board. A significant difference (*P* < 0.05) was observed in the load of* Staphylococcus *spp. in cleaning cloth which was dirty used by the vendor when compared to the load of* Staphylococcus *in neat cloth used by the vendor. The pathogenic bacteria population was significantly higher in south circle because demographically this circle belongs to the old part of the city wherein the density of the population is high and most of the population was inhabited by lower income group which may be one of the factors contributing to poor hygienic practices.

## 5. Conclusions

The present study revealed that the potential hazard of street vended salad vegetables may be due to handling practices of vendors and poor hygienic conditions in which they are sold. Salads can be a potential source of* Salmonella *infection among the consumers. A simple thorough washing of vegetables with safe running water before further processing reduces the risk of microbiological hazards. There is a need to provide basic training on food hygiene to food vendors to ensure food safety and also create consumer awareness of consuming improperly processed salads as one of the possible sources of microbial hazards.

## Figures and Tables

**Table 1:** Incidence of foodborne pathogens in salads given along with street foods in Hyderabad.

Pathogen	Carrot (*n* = 53)	Onion (*n* = 110)
*Staphylococcus *	39 (73.6%)	62 (56.4%)
*Salmonella *	31 (58.5%)	50 (45.5%)
*Yersinia *	36 (67.9%)	26 (23.6%)

**Table 2:** Mean concentration ranges of *Staphylococcus*, *Salmonella*, and *Yersinia *in salad samples.

Pathogens	Carrot (min–max)	Mean	Onion (min–max)	Mean
*Staphylococcus *	2.0–5.2 (10^2^–10^5^)	4	2.0–5.0 (10^2^–10^5^)	3.2
*Salmonella *	3.0–5.4 (10^3^–10^5^)	4.4	2.0–5.4 (10^2^–10^5^)	3.9
*Yersinia *	3.3–5.4 (10^3^–10^5^)	4.5	2.9–4.8 (10^2^–10^5^)	3.9

*Range of microbial counts (log⁡10 cfu/g).

**Table 3:** Salad handling practices and preparation and storage practices of 53 street food venders in Hyderabad city.

Practices	Frequency of responses (*n* = 53) %
Vegetables from	
(a) Market	49 (92.5)
(b) Shop	4 (7.5)
Vegetables brought in	
(a) Polythene bag	23 (43.4)
(b) Gunny bags	30 (56.6)
Frequency	
(a) Once in a day	8 (15.1)
(b) Once in 2 days	15 (28.3)
(c) Once in 5 days	16 (30.2)
(d) Once in a week	14 (26.4)
Storage	
(a) House	30 (56.6)
(b) Stall	23 (43.4)
Storage before cutting	
(a) Table	10 (18.9)
(b) Bowl	11 (20.8)
(c) Bucket	12 (22.6)
(d) Refrigerator	10 (18.9)
(e) Others	10 (18.9)
Wash vegetables	
(a) Yes	1 (1.9)*
(b) No	52 (98.1)
Peeling vegetables	
(a) Yes	23 (43.4)
(b) No	30 (56.6)
Peeled with	
(a) Peeler	1 (1.9)
(b) Knife	22 (41.5)
(c) Not peeled	30 (56.6)
Cutting vegetable	
(a) Knife	49 (92.5)
(b) Vegetable cutter	3 (5.7)
(c) Grater	1 (1.9)
Status of knife	
(a) Clean-dry	15 (28.3)
(b) Wet-dirty	38 (71.7)
Storage after cutting	
(a) Open place	29 (54.7)
(b) Container	24 (45.3)
Selling of non-veg. food	
(a) Yes	6 (11.3)^~^
(b) No	47 (88.6)
Frequent washing of knife	
(a) Yes	6 (11.3)
(b) No	47 (88.7)
Cutting and chopping place	
(a) Wooden	48 (90.6)
(b) Stainless table	5 (9.4)
Status of cleaning cloth	
(a) Neat	5 (9.4)
(b) Dirty	48 (90.6)
Does vendor wear gloves?	
(a) No	53 (100)
Frequent washing of hands	
(a) Yes	3 (5.7)
(b) No	50 (94.3)
Status of his nails	
(a) Neat	21 (39.6)
(b) Dirty	32 (60.4)
Wounds	
(a) Cut	6 (11.3)
(b) Wound	1 (1.9)
(c) No wound	46 (86.8)
Status of nails	
(a) Cut	21 (39.7)
(b) Uncut	32 (60.3)
Neatly combed	
(a) Yes	16 (30.2)
(b) No	37 (69.8)
Uniform	
(a) Neat	12 (22.6)
(b) Dirty	41 (77.4)
Status of premises	
(a) Near to the water drain	9 (17)
(b) Adjacent to road	34 (64.2)
(c) Near public toilet	3 (5.7)
(d) Near municipal garbage	4 (7.5)
(e) Others	3 (5.7)
Waste disposal	
(a) Garbage bin	40 (75.5)
(b) Plastic bag	12 (22.6)
(c) Outside	1 (1.9)
Stray dogs and cats in vicinity	
(a) Yes	53 (100)
Rodent droppings	
(a) Yes	31 (58.5)
(b) No	22 (41.5)
Insect exposure	
(a) Yes	51 (96.2)
(b) No	2 (3.8)

*Washing is done with still water. ^~^Use same knife to cut vegetables and meat.

**Table 4:** HACCP analysis of *Salmonella* (cfu/g) contamination in salads.

	Salad sample	Hand wash	Knife swab	Chopping board swab
Vendor 1	ND	1 × 10^3^	ND	ND
Vendor 2	7 × 10^4^	12 × 10^4^	ND	ND
Vendor 3	3 × 10^5^	6 × 10^3^	18 × 10^3^	5 × 10^4^
Vendor 4	ND	6 × 10^3^	ND	ND
Vendor 5	3 × 10^5^	ND	4 × 10^3^	3 × 10^5^
Vendor 6	ND	1 × 10^4^	ND	1 × 10^3^

ND: not detected.
